# The Finsen Light

**Published:** 1902-10-04

**Authors:** 


### THE FINSEN LIGHT.

E. G. Benwell, Secretary the Eversfield Hospital, St. Leonards-on-Sea, writes: May I draw your attention to the fact that the Finsen Light has been installed in this hospital, and that we are glad to receive patients for this treatment?

Miss Edith M. Vizard, lady superintendent, the Royal Alexandra Hospital, Rhyl, writes: "Having seen in The Hospital that you will be glad to hear of hospitals where the Finsen light has been installed, I write to say that it is in use here, and also the X-rays.

